# Loss of the 14-3-3σ is essential for LASP1-mediated colorectal cancer progression via activating PI3K/AKT signaling pathway

**DOI:** 10.1038/srep25631

**Published:** 2016-05-09

**Authors:** Ziyun Shao, Yanjun Cai, Lijun Xu, Xueqing Yao, Jiaolong Shi, Feifei Zhang, Yuhao Luo, Kehong Zheng, Jian Liu, Fengliu Deng, Rui Li, Lanzhi Zhang, Hui Wang, Mingyi Li, Yanqing Ding, Liang Zhao

**Affiliations:** 1 Department of Pathology, Nanfang Hospital, Southern Medical University, Guangzhou, China; 2 Department of Gerontology, Guangzhou General Hospital of the Guangzhou Military Command of the People’s Liberation Army (PLA), Guangzhou, China; 3 The Department of General Surgery, Guangdong General Hospital, Guangdong Academy of Medical Science, Guangzhou, Guangdong, China; 4 Department of General Surgery, Zhujiang Hospital, Southern Medical University, Guangzhou, China; 5 Department of Medical Oncology, Affiliated Tumor Hospital of Guangzhou Medical University, Guangzhou, China; 6 Radiotherapy Department, Affiliated Tumor Hospital of Guangzhou Medical University, Guangzhou, China; 7 Department of Pathology, School of Basic Medical Sciences, Southern Medical University, Guangzhou, China

## Abstract

LIM and SH3 protein 1 (LASP1) can promote colorectal cancer (CRC) progression and metastasis, but the direct evidence that elucidates the molecular mechanism remains unclear. Here, our proteomic data showed that LASP1 interacted with 14-3-3σ and decreased the expression of 14-3-3σ in CRC. Deletion of 14-3-3σ was required for LASP1-mediated CRC cell aggressiveness. *In vitro* gain- and loss-of-function assays showed that 14-3-3σ suppressed the ability of cell migration and decreased the phosphorylation of AKT in CRC cells. We further observed clearly co-localization between AKT and 14-3-3σ in CRC cells. Treatment of PI3K inhibitor LY294002 markedly prevented phosphorylation of AKT and subsequently counteract aggressive phenotype mediated by siRNA of 14-3-3σ. Clinically, 14-3-3σ is frequently down-regulated in CRC tissues. Down-regulation of 14-3-3σ is associated with tumor progression and poor prognosis of patients with CRC. Multivariate analysis confirmed low expression of 14-3-3σ as an independent prognostic factor for CRC. A combination of low 14-3-3σ and high LASP1 expression shows a worse trend with overall survival of CRC patients. Our research paves the path to future investigation of the LASP1-14-3-3σ axis as a target for novel anticancer therapies of advanced CRC.

Colorectal cancer (CRC) is one of the most common malignancies worldwide and the leading cause of cancer deaths^1^. The incidence of colorectal cancer is increasing in China. Despite recent advances in the diagnosis and therapy of colorectal cancer, the general survival rate of patients with colorectal cancer has not improved. Clinically, metastasis is still the main cause of mortality and poor prognosis^2^,^3^, yet there is lack of effective strategies for its management. The molecular mechanisms underlying colorectal cancer metastasis are not quite clear till date.

LIM and SH3 protein 1 (LASP1) was initially identified from metastatic axillary lymph nodes of breast cancer patients. The up-regulated expression of LASP1 has been found in several types of cancers^4^,^5^,^6^. In previous studies, we illustrated that miR-1 and miR-133a could inhibit LASP1 expression by directly binding with its 3’UTR region in CRC cells^7^. Epigenetic silencing of miR-1 and miR-133a by promotor hypermethylation resulted in over-expression of LASP1 in CRC tissues. An over-expression of LASP1 was required for TGFβ-mediated epithelial-mesenchymal transition (EMT) and aggressive phenotypes of cancer cells, thereby promoting cancer progression^8^,^9^. Clinically, the expression of this protein was closely correlated with lymph node status, thereby improving the overall survival rates of patients with CRC. These results indicated that LASP1 might be a promising molecule that could be used in developing treatments for patients with advanced CRC. Presently, we do not have any direct evidence that elucidates the molecular mechanism of LASP1 in CRC metastasis.

In this study, we identified 14-3-3σ as a LASP1-modulated proteins using proteomic strategy. Furthermore, we investigate the involvement of 14-3-3σ in LASP1-mediated CRC metastasis by rescue experiments. We also determined the involvement of LASP1 in activation of PI3K/AKT signaling pathway in CRC cell lines while examining mechanisms underlying its effect in CRC. Finally, clinical significance of 14-3-3σ and its relationship with LASP1 in CRC tissues were analyzed. We wanted to deepen our understanding of CRC metastasis and provide the experimental basis for targeted treatment of patients with advanced CRC.

## Materials and Methods

### Cell culture and inhibitor treatment

CRC cell lines LS174t, RKO, HT29, HCT116, SW480, and SW620 were obtained from the Cell Bank of the Chinese Academy of Sciences (Shanghai, China) and maintained as previously described^8^. All cells were authenticated by short tandem repeat (STR) profiling before receipt and were propagated for less than 6 months after resuscitation. Additionally, a human CRC cell subline with unique liver metastatic potential, designated as SW480/M5, was established in our laboratory^10^ and used in the analysis. All the cells were cultured in RPMI 1640 (Hyclone; Logan, Utah, USA) supplemented with 10% fetal bovine serum (FBS) (Gibco-BRL, Invitrogen; Paisley, UK) at 37^o^C with a humidity of 5% CO_2_. For inhibitor treatment, 10 mmol/L PI3K inhibitor LY294002 (Cell Signal Technology, Danvers, MA) was added in the cultured cells every 2 days.

### Tumor tissue samples

Fresh primary CRC specimens and paired noncancerous colorectal tissue were provided by the Tumor Tissue Bank of Nanfang Hospital. In each case, a diagnosis of primary CRC had been made, and the patient had undergone elective surgery for CRC in Nanfang Hospital between 2007 and 2010. The pathological diagnosis was made in the Department of Pathology of Nanfang Hospital of Southern Medical University. The study was approved by the Ethics Committee of Southern Medical University and all aspects of the study comply with the Declaration of Helsinki. Ethics Committee of the Southern Medical University specifically approved that not informed consent was required because data were going to be analyzed anonymously.

### Western blot analysis

Protein expression was assessed by immunoblot analysis of cell lysates (20–60 μg) in RIPA buffer in the presence of rabbit antibodies to β-actin, GAPDH (1:500; Santa Cruz, California, USA); mouse antibody to 14-3-3σ (1:1000; Sigma, St. Louis, MO); rabbit antibodies to p-AKT(Ser473), AKT (1:1000; CST, Danvers, MA) and mouse antibody to LASP1 (1:1000; Millipore, Billerica, MA).

### Statistical analysis

Data were analyzed using SPSS version 19.0 software (SPSS; Chicago, USA). The Student *t*-test and the one-way ANOVA test were carried out for qRT-PCR. Significance of correlation between the expression of 14-3-3σ and histopathological factors were determined using Pearson’s chi-squared (χ^2^) test. The correlation between 14-3-3σ and LASP1 was determined using the Spearman rank correlation test. Kaplan-Meier plots were used to estimate the prognostic relevance of 14-3-3σ in univariate analysis. Multivariate analysis was performed by applying Cox proportional hazards test. Statistical significance was established at *P* < 0.05.

## Results

### LASP1 negatively regulates 14-3-3σ expression by protein interaction

**Figure 1:**
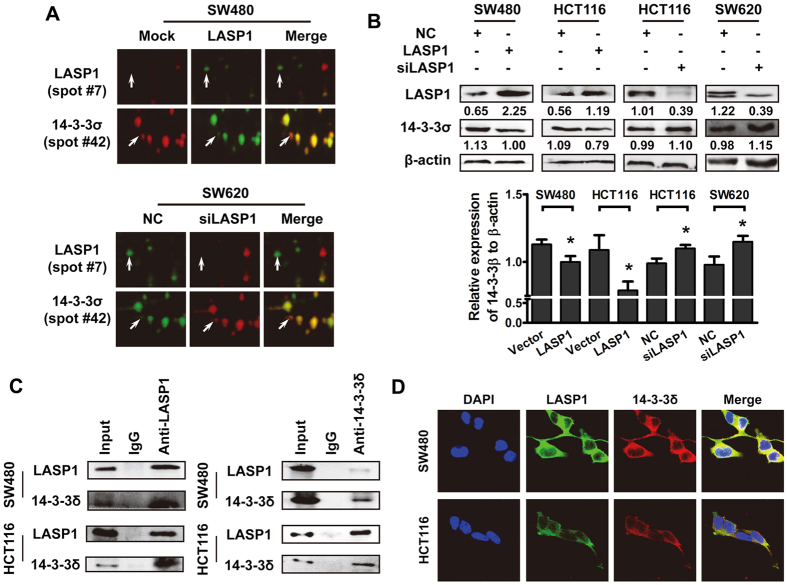
LASP1 negatively regulates 14-3-3σ expression by protein interaction. (**A**) DIGE was performed to screen the differentially expressed proteins in SW480/LASP-1 cells or SW620 cells that were transfected with LASP-1 siRNA and control cells. The enlarged images of two differentially expressed protein spots in DIGE analysis were shown. The protein spots are indicated (white arrows). (**B**) Western blot was performed to detect the expression of 14-3-3σ and LASP1 protein in indicated cells. The immunosignal was quantified using densitometric scanning software, and relative protein abundance was determined by normalisation with levels of β-actin. Each bar represented the mean ± SD. The results were reproduced in three independent experiments. The asterisk (*) indicates P < 0.05. The asterisk (**) indicates P < 0.01. (**C**) Endogenous interaction between 14-3-3σ and LASP1 in CRC cells. Cells were lysed and purified by anti-LASP1 or anti-14-3-3σ affinity gel; protein pellets were analyzed by western blot with anti-LASP1 or anti-14-3-3σ. (**D**) The subcellular localization of 14-3-3σ and LASP1 in indicated cells was assessed by immunofluorescence staining. The full-length blots/gels are presented in Supplementary Fig. S3.

Using 2-D difference gel electrophoresis (2-D DIGE)-based proteomic strategy, one of the candidates LASP1-modulated proteins was identified as 14-3-3σ, which was negatively correlated with LASP1 expression (Fig. 1A). Western blot analysis showed that decreased expression of 14-3-3σ was observed in LASP1-overexpressing SW480 and HCT116 cells, meanwhile increased expression of 14-3-3σ was detected in LASP1-silenced SW620 and HCT116 cells (Fig. 1B), which are consistent with proteomic results.

To address the regulatory mechanism of LASP1, we detected the effect of LASP1 on transcriptional activation of 14-3-3σ. The results showed that LASP1 did not affect the expression of 14-3-3σ mRNA (Supplementary Fig. S1). Interestingly, we observed clearly co-localization between LASP1 and 14-3-3σ in SW480 and HCT116 cells (Fig. 1C). The protein interaction was also validated by co-Immunoprecipitation (Co-IP) assay in protein extraction of SW480 and HCT116 cells (Fig. 1D). This protein interaction, however, did not influence the expression of LASP1 protein (Supplementary Fig. S2)

### 14-3-3σ suppresses cell migration via inhibiting phosphorylation of AKT

**Figure 2:**
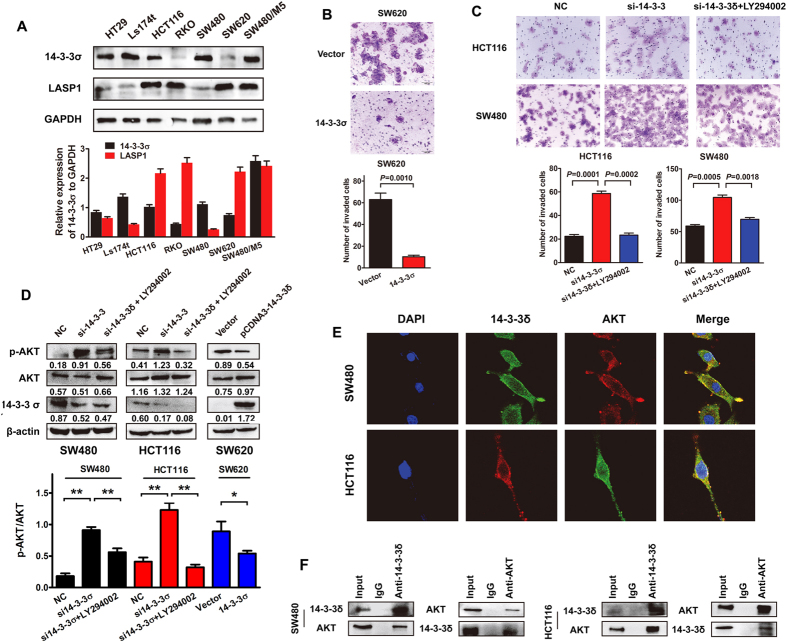
14–3–3σ suppresses cell migration via inhibiting phosphorylation of AKT. (**A**) Western blot analysis for the expression of 14-3-3σ in CRC cell lines. Immunosignals were quantified by densitometric scanning. 14-3-3σ expression in the individual cells was calculated as 14-3-3σ expression relative to GAPDH expression. Data are means + SD from three independent experiments. (**B**) The representative figures and data of transwell assay for SW620 cells were transfected with 14-3-3σ vector. Each bar represented the mean ± SD. The results were reproduced in three independent experiments. (**C**) The representative figures and data of transwell assay for SW480 and HCT116 cells co-infected with 14-3-3σ siRNA and PI3K inhibitor LY294002. (**D**) Western blot analysis of phosphorylated AKT in indicated cells transfected with 14-3-3σ vector or co-infected with 14-3-3σ siRNA and PI3K inhibitor LY294002. The immunosignal was quantified using densitometric scanning software, and relative protein abundance was determined by normalisation with total levels of AKT. Each bar represented the mean ± SD. The results were reproduced in three independent experiments. The asterisk (*) indicates P < 0.05. The asterisk (**) indicates P < 0.01. (**E**) The subcellular localization of 14-3-3σ and AKT in indicated cells was assessed by immunofluorescence staining. (**F**) Endogenous interaction between 14-3-3σ and AKT in CRC cells. Cells were lysed and purified by anti-AKT or anti-14-3-3σ affinity gel; protein pellets were analyzed by western blot with anti-AKT or anti-14-3-3σ. Representative figures were shown. The results were reproduced in 3 independent experiments. The full-length blots/gels are presented in Supplementary Fig. S6.

We detected the endogenous expression of 14-3-3σ and LASP1 protein in all seven CRC cell lines. Accept for SW480/M5, we observed a negative correlation between two protein in other CRC cells (R = −0.393, *P* = 0.383). Interestingly, a significantly decreased expression of 14-3-3σ was found in SW620 cells derived from lymph node metastasis, compared with SW480 cells derived from the primary lesion in the same patient (Fig. 2A). *In vitro* loss- and gain-of-function assays were carried out to investigate the effect of 14-3-3σ on CRC cellular biological behaviors. Transwell assay showed that exogenous introduction of 14-3-3σ resulted in decreased ability of cell migration, invasion and motility in SW620 cells with a relatively low 14-3-3σ expression level (Fig. 2B; Supplementary Fig. S4). On the contrary, siRNA-mediated depletion of endogenous 14-3-3σ expression enhanced migratory ability in SW480 and HCT116 cells (Fig. 2C).

To uncover the mechanism underlying tumor suppressor induced by 14-3-3σ, we performed western blot analysis to detect the phosphorylation level of AKT. The results showed that 14-3-3σ suppressed the phosphorylation of AKT in CRC cells (Fig. 2D). Meanwhile, treatment of PI3K inhibitor LY294002 markedly prevented phosphorylation of AKT and subsequently counteract aggressive phenotype mediated by siRNA of 14-3-3σ (Fig. 2D). Furthermore, we observed clearly co-localization between AKT and 14-3-3σ in SW480 and HCT116 cells (Fig. 2E). The protein interaction was also validated by co-IP assay in protein extraction of SW480 and HCT116 cells (Fig. 2F). The corresponding interaction between 14-3-3σ and p-AKT were also observed in SW480 cells (Supplementary Fig. S5).

### Loss of 14-3-3σ is essential for LASP1-mediated cell aggressiveness

**Figure 3:**
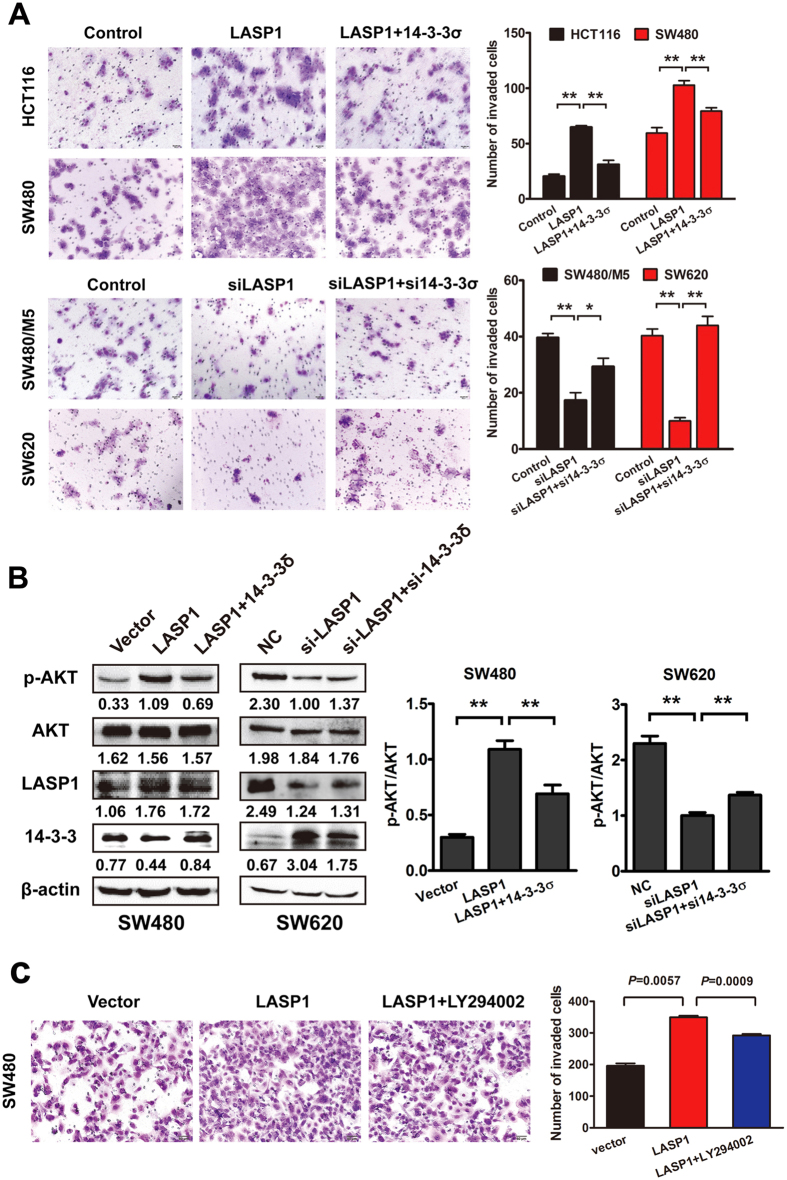
Loss of 14-3-3σ is essential for LASP1-mediated cell aggressiveness. (**A**) Representative figures and data of transwell assay for indicated cells. (**B**) Western blot analysis of phosphorylated AKT in indicated cells co-infected with transfected with 14-3-3σ and LASP1 vector or siRNA. The immunosignal was quantified using densitometric scanning software, and relative protein abundance was determined by normalisation with total levels of AKT. Each bar represented the mean ± SD. The results were reproduced in three independent experiments. The asterisk (*) indicates P < 0.05. The asterisk (**) indicates P < 0.01. The full-length blots/gels are presented in Supplementary Fig. S7.

To address the pivotal role of 14-3-3σ in LASP1-mediated cell aggressive phenotype, we performed the rescued experiment to detect activation of PI3K/AKT signaling pathway and aggressive capacity of CRC cells. In agreement with our previous study, LASP1 could promote migratory captivity of CRC cells via activating PI3K/AKT signaling pathway. Restoring expression of 14-3-3σ weakened cell migration induced by LASP1 in CRC cells, whereas depletion of 14-3-3σ recovered aggressive capacity of CRC cells suppressed by siRNA of LASP1 (Fig. 3A). Western blot analysis confirmed that loss of 14-3-3σ was required for PI3K/AKT signaling pathway activated by LASP1 (Fig. 3B). Furthermore, PI3K inhibitor LY294002 could partly neutralize cell migration promoted by LASP1, suggesting the essential role of AKT activation in LASP1-mediated aggressive phenotype (Fig. 3C).

### 14-3-3σ is frequently down-regulated in CRC tissues

**Figure 4:**
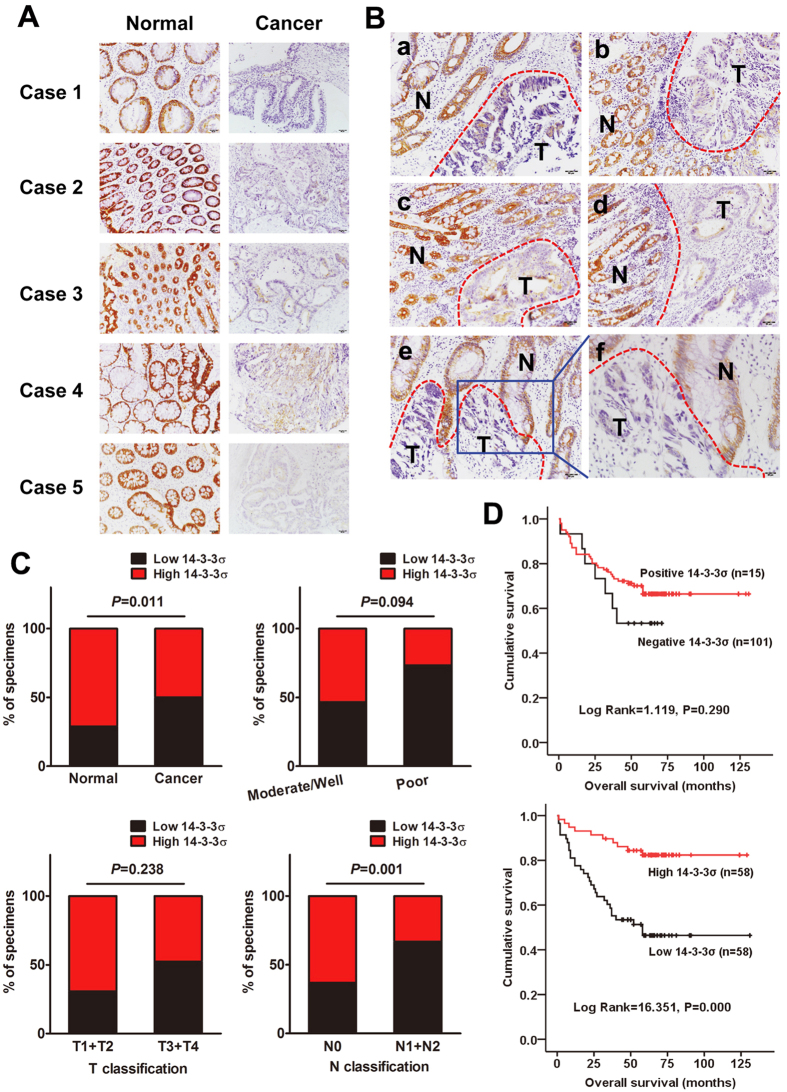
14–3–3σ is frequently down-regulated in CRC tissues. (**A**) IHC analysis of 14-3-3σ protein expression in CRC tissues (T) and adjacent non-tumor tissues (N). (**B**) IHC analysis of 14-3-3σ protein expression in the junction of benign and malignant colorectal lesions. In the bottom row, right picture (f) corresponded with high magnification of left picture (e). Scale bars were shown in the lower right corner of each picture. N, adjacent non-tumor tissues. T, CRC tissues. (**C**) Graphical illustration of statistical 14-3-3σ distribution in 116 cases of CRC tissues. 14-3-3σ is significantly higher in CRC than adjacent non-tumorous tissue. The high expression of 14-3-3σ is more frequently found in CRC with lymph node metastasis (N1 + N2) than in CRC without lymph node metastasis (N0). (**D**) Kaplan-Meier survival curves and univariate analyses (log-rank) for CRC patients with positive expression versus negative expression or low expression versus high expression of 14-3-3σ in 116 cases of CRC tissues.

To evaluate expression and subcellular localization of 14-3-3σ protein, we performed immunohistochemical assay in 52 and 116 paraffin-embedded, archival normal colorectal mucosa and CRC tissues, respectively. Positive signals of 14-3-3σ were predominantly detected in the cytoplasm of benign and malignant epithelial cells. As shown in Fig. 4A, decreased expression of 14-3-3σ was frequently found in CRC tissues compared with adjacent non-tumorous tissues tested. Interestingly, deletion of 14-3-3σ expression was also observed in carcinogenetic lesions surrounding normal mucosa, especially in malignant cells derived from benign gland (Fig. 4B).

Immunohistochemistry clearly allowed to localise 14-3-3σ expression in 92.3% (48 of 52) of adjacent non-tumorous tissues tested. As compared to these non-tumorous tissues, we observed 14-3-3σ protein expression in 87.1% (101 of 116) of all CRC samples (*P* = 0.433). According to reclassification as Supplementary Materials and Methods, 14-3-3σ was evaluated as high expression in 50.0% (58 of 116) of tumor samples, compared with 71.2% (37 of 52) of adjacent non-tumorous samples (*P* = 0.011) (Supplementary Table S3).

### Down-regulation of 14-3-3σ is associated with tumor progression and poor prognosis of patients with CRC

To evaluate the clinical relevance of 14-3-3σ expression, we analyzed its relationship with pathological features. As shown in Supplementary Table S3, expression of 14-3-3σ was negatively associated with N classification (lymph node metastasis) of patients with CRC (*P* = 0.001) (Fig. 4C). Kaplan-Meier survival curves revealed a significant trend towards poorer survival for patients whose primary tumors showed low 14-3-3σ expression, compared with those patients whose primary tumors showed high 14-3-3σ expression (Log Rank = 16.351, *P* = 0.000; Fig. 4D). Furthermore, multivariate analysis confirmed low expression of 14-3-3σ, including gender, N classification and M classification, as independent prognostic factors for CRC (Supplementary Table S4).

### 14-3-3σ expression is inversely correlated with LASP1 expression in CRC tissues

**Figure 5:**
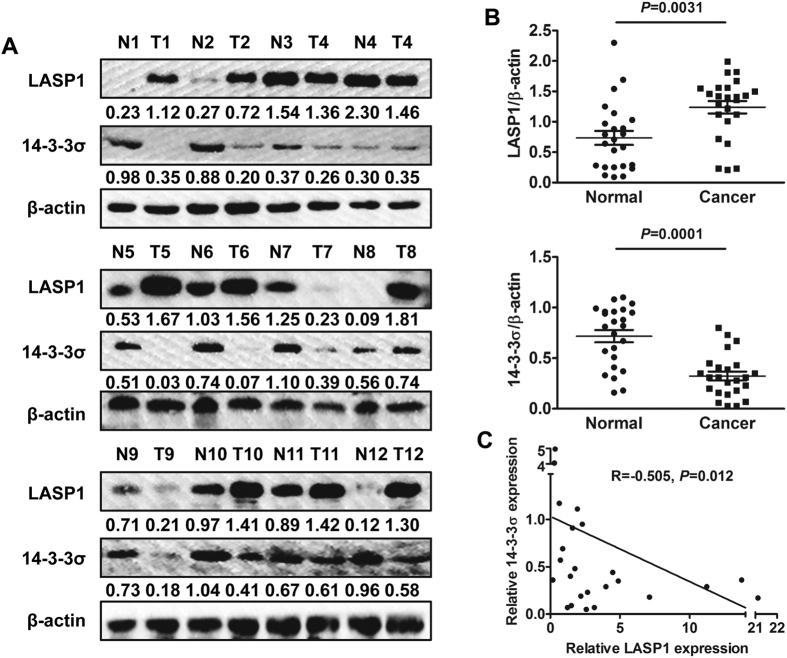
14–3–3σ expression is inversely correlated with LASP1 expression in CRC tissues. (**A**) Western blot analysis of LASP1 and 14-3-3σ in CRC tissues (T) and adjacent non-tumor tissues (N). (**B**) The expression of LASP1 and 14-3-3σ in the paired CRC tissues and matched normal tissues. The immunosignal was quantified using densitometric scanning software, and the relative protein abundance was determined by normalization with β-actin. (**C**) A statistically significant inverse correlation between 14-3-3σ and LASP1 protein was observed in CRC specimens. The full-length blots/gels are presented in Supplementary Fig. S8.

**Figure 6:**
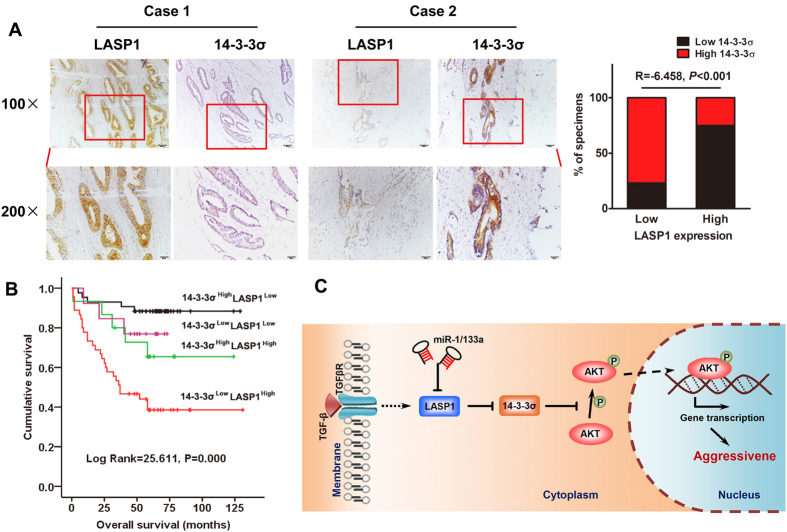
LASP1 and 14-3-3σ corporately contribute to CRC aggressiveness. (**A**) Paraffin-embedded CRC sections were stained with anti-14-3-3σ or anti-LASP1 antibodies. Visualizations of two representative cases were shown. Lower panels are corresponding high magnification of upper panels. The low expression of 14-3-3σ is more frequently found in CRC cases with LASP1 overexpression. (**B**) Prognostic significance of a combination of 14-3-3σ and LASP1 assessed using Kaplan-Meier survival and univariate analyses (log-rank) for CRC patients in 116 cases. (**C**) A hypothetical model illustrating that loss of the 14-3-3σ is essential for LASP1-mediated colorectal cancer progression via activating PI3K/AKT signaling pathway.

Western blot technique was used to detect LASP1 and 14-3-3σ expression in 24 cases of fresh CRC tissues (Fig. 5A). The results suggested that 14-3-3σ expression was mainly lower in CRC tissues than in paired non-cancerous colorectal tissues (Fig. 5B; *P* = 0.0031) and had a negative correlation with LASP1 expression (Fig. 5C; R = −0.505, *P* = 0.012). Similarly, immunohistochemical results from a larger population-based cohort study showed that relatively low expression of 14-3-3σ was frequently observed in CRC samples with LASP1 overexpression (Fig. 6A). There was also a negative correlation between 14-3-3σ and LASP1 expression (R = −6.458, *P* < 0.001).

Based on the above data, we then tested a combination of 14-3-3σ and LASP1 and studied its predictive value for overall survival. Intriguingly, patients with high LASP1 and low 14-3-3σ had significantly worse outcome, and patients with low LASP1 and high 14-3-3σ had better outcome, indicating the opposing effects of LASP1 and 14-3-3σ on CRC patient survival (Fig. 6B).

## Discussion

Numerous evidences have demonstrated that LASP1 was significantly overexpressed in various different cancer entities and contributed to tumor aggressiveness hinting to its potential value as a cancer biomarker^11^,^12^. LASP1 is comprised of one N-terminal LIM and one C-terminal SH3 domain, allowing interaction with various cytoskeletal and signaling proteins^11^. However, the detailed molecular mechanism underlying LASP1-mediated CRC progression remains unknown. To combine proteomic analysis with western blot validation, 14-3-3σ was identified as a candidate LASP1-modulated protein. LASP1 interacted with 14-3-3σ and negatively regulated the expression of 14-3-3σ. LASP1-14-3-3σ protein complex may contribute to the stability of 14-3-3σ through hindering its’dimerization to form a more rigid structure^13^. Loss or gain of 14-3-3σ partially neutralize the effect of LASP1 on cell migration in CRC cells, suggesting that 14-3-3σ is a directly downstream effector of LASP1 through which LASP1 exerts its pro-migratory effects on CRC cells.

14-3-3σ is also called stratifin, a member of 14-3-3 family with a highly conserved group of proteins constituted by sever isoforms. It was originally characterized as a human mammary epithelium marker 1 (HME1) and identified as a tumor suppressor gene, which contributes to cancer development^14^,^15^. Increasing evidences showed that 14-3-3σ was significantly decreased or lost in several kinds of solid tumors^12^, which are consistent with our present data in clinical samples tested. However, contradictory with the tumor suppressor role of 14-3-3σ, its overexpression was frequently observed in pancreatic^16^ and gastric cancer^17^. Thus, the biological role of 14-3-3ơ in various types of human cancers varies depending on specific tumor type. Many studies have pointed out the importance of 14-3-3ơ promoter hypermethylation in silencing of 14-3-3ơ gene^18^,^19^. In terms of CRC, however, the hypermethylation of the 14-3-3ơ gene was an uncommon event^20^. We, therefore, considered that regulation of LASP1, but not 14-3-3ơ promoter hypermethylation, probably play a significant role in the down-regulation of 14-3-3ơ expression, at least in human CRC.

Our current study revealed the associated of 14-3-3ơ expression with lymph node in CRC, suggesting that absent 14-3-3ơ expression can be considered as such an indicator of the propensity of metastasis. Furthermore, overexpression of 14-3-3ơ was able to inhibit *in vitro* migratory ability of CRC cells, further supporting that 14-3-3ơ is involved in CRC metastasis. Additionally, the previous study found that 14-3-3ơ interacted with constitutive photomorphogenic 1 (COP1), a p53-targeting E3 ubiquitin ligase, and controlled COP1 protein stability after DNA damage. COP1 expression promoted cell proliferation, cell transformation, and tumor progression, manifesting its role in cancer promotion, whereas 14-3-3ơ negatively regulated COP1 function and prevented tumor growth in a mouse xenograft model of human cancer^21^. Our results, together with this report, suggest 14-3-3ơ functions as a potential tumor suppressor gene in CRC progression.

In our previous study, LASP1 activated PI3K/AKT signaling pathway by regulating the protein phosphorylation level^9^, which were recognized as pivotal link of cancer progression. However, the exact mechanism has not been fully established. Over hundred small molecules interact with 14-3-3 in a phosphorylation-dependent manner^22^. These proteins include protein kinases, receptor proteins, scaffolding molecules and proteins involved in cell cycle control and apoptosis^23^,^24^. However, a few proteins interact with 14-3-3 in a phosphorylation-independent manner such as LASP1, identified in our study, and Bax^25^. In breast cancer, AKT activation is diminished by the expression of a p53-inducible protein, 14-3-3ơ^26^. Evidence has been provided that 14-3-3ơ binds and inhibits AKT. Low expression of 14-3-3ơ in human primary breast cancers correlates with AKT activation^27^,^28^. Structure studies have supported such a concept: 14-3-3ơ has unique amino acids (Met202, Asp204, and His206) that may be responsible for binding particular ligands^29^,^30^. In the present study, we also demonstrated that 14-3-3ơ could interact with AKT and counteract LASP1-dependent activation of AKT kinase in CRC aggressiveness, suggesting 14-3-3ơ as a molecular regulator of AKT and as a potential anticancer agent for AKT-activated cancers.

Despite recent progress towards elucidating the functions of 14-3-3ơ in cell cycle regulation, apoptosis, migration, and differentiation, the prognostic value in patients with cancer has not been widely studied. Loss of 14-3-3ơ has been shown to be an independent predictor for worse overall survival in patients with endometrial^19^, nasopharyngeal^31^ and esophageal^32^ cancer. In contrast, gain of 14-3-3ơ was seen as an independent predictor for poor survival rate in patients with breast^16^ and gastric^17^ cancer. Although a study initially proposed the predictive value of 14-3-3ơ overexpression in patients with CRC^33^, later and more extensive studies showed that loss of 14-3-3ơ contributed to CRC aggressiveness^21^,^34^. In our current study, low 14-3-3ơ expression was related to poor prognosis and might be served as an independent factor in patients with CRC. In view of the opposing effects of LASP1 and 14-3-3σ on CRC patient survival, we combined both of proteins to evaluate its predictive value. The results showed that patients with high LASP1 and low 14-3-3σ had significantly worse outcome, and patients with low LASP1 and high 14-3-3σ had better outcome.

In summary, the data presented here provide the suppressive role of 14-3-3σ in LASP1-assoicated CRC metastasis. LASP1, a downstream effector of TGFβ, could negatively regulate expression of 14-3-3σ via protein interaction. Silencing of 14-3-3σ plays an essential role in LASP1-mediated cell migration through binding AKT and inhibiting activity of PI3K/AKT signaling pathway (Fig. 6C). Clinical sample studies indicate that 14-3-3σ is associated with CRC metastasis and poor prognosis of patients and is inversely correlation with LASP1 expression. A combination of low 14-3-3σ and high LASP1 expression shows a better trend with overall survival of CRC patients. Therefore, our research paves the path to future investigation of the LASP1-14-3-3σ axis as a target for novel anticancer therapies of advanced CRC.

## Additional Information

**How to cite this article**: Shao, Z. *et al*. Loss of the 14-3-3σ is essential for LASP1-mediated colorectal cancer progression via activating PI3K/AKT signaling pathway. *Sci. Rep*. **6**, 25631; doi: 10.1038/srep25631 (2016).

## Supplementary Material

Supplementary Information
