# Supplementary material for: Loss of the 14-3-3σ is essential for LASP1-mediated colorectal cancer progression via activating PI3K/AKT signaling pathway
Source: Sci Rep. 2016 May 9;6:25631. doi: 10.1038/srep25631 (PMC4860602; doi:10.1038/srep25631)
Supplement: Supplementary Information [file srep25631-s1.pdf]

Supplementary Information

Loss of the 14-3-3σ is essential for LASP1-mediated colorectal cancer progression via activating PI3K/AKT signaling pathway

Ziyun Shao[1], Yanjun Cai[2], Lijun Xu[1], Xueqing Yao[3], Jiaolong Shi[1], Feifei Zhang[1], Yuhao Luo[1], Kehong Zheng[3], Jian Liu[1], Fengliu Deng[1], Rui Li[1], Lanzhi Zhang[1], Hui Wang[5], Mingyi Li[6], Yanqing Ding[1,7], Liang Zhao[1,7]

Corresponding author at: Department of Pathology, Nanfang Hospital, Southern Medical University, Guangzhou, China. E-mail address: liangsmu@foxmail.com (L. Zhao) and dyqsmu@sina.com (Y. -Q. Ding).

Supplementary Materials and Methods

RNA isolation, reverse transcription, and quantitative real-time PCR

Total RNA was extracted using Trizol reagent (Invitrogen). Total RNA was polyadenylated and underwent reverse transcription using PrimeScript™ RT Master Mix (TaKaRa, Dalian, China). Real-time PCR was carried out using a SYBR® Premix Ex TaqTM II (TaKaRa, Dalian, China) on an ABI 7500HT system. GAPDH was used as an endogenous control. All samples were normalized to internal controls, and fold changes were calculated through relative quantification (2-ΔΔCT). The primers used are shown in Supplementary Table S1

Immunofluorescence (IF)

Cells were cultured on coverslips overnight, fixed with 4% paraformaldehyde for 20 min and treated with 0.25% Triton X-100 for 10 min. After blocking in 10% normal blocking serum at room temperature for 10 min, slides were incubated with rabbit anti-14-3-3σ (1:200; Sigma, St. Louis, MO), rabbit antibodies to AKT (1:200; Santa Cruz, California, USA) and mouse anti-LASP1(1:100; Millipore, Billerica, MA) antibodies at 4°C overnight followed by washing with PBS three times. Coverslips were then incubated with fluorescein isothiocyanate (FITC)-conjugated and Texas Red (TR)-conjugated antibodies (1:120; Santa Cruz) for 30 min at room temperature, and then stained with 6-diamidino-2-phenylindole (DAPI; Invitrogen).

Proteomic analysis

Conventional 2-D electrophoresis analysis and MS identification were performed

as previously described. [1] For 2-D difference gel electrophoresis (2-D DIGE), the proteins were labelled with fluorescent cyanine dyes (GE Healthcare, Milwaukee, Wisconsin, USA) following the manufacturer's instructions. In brief, 50 mg of extracted protein to be compared was labelled with 400 pmol Cy3 or Cy5, while 400 pmol Cy2 was employed to label 50 mg of internal standard protein of each sample at an equal amount. The labelling was performed on ice in the dark for 30 min, and then quenched with 1 ml of 10 mM lysine (Sigma) for 10 min. A 50 mg aliquot of Cy3- and Cy5-labelled samples was combined before mixing with 50 mg of Cy2-labelled internal standard. The 2× sample buffer with an equal volume (8 M urea, 2 M thiourea, 4% CHAPS, 2% Bio-lyte, pH 4-7, 130 mM dithiothreitol (DTT)) was added to the sample, and the final volume was adjusted to 450 ml with rehydration buffer (8 M urea, 4% CHAPS, 1% Biolyte, pH 4e7, 40 mM DTT). The proteins were applied to IPG (immobilised pH gradient) strips (pH 4-7, 24 cm) and focused on an IPGphor (GE Healthcare). Focused IPG strips were equilibrated, and then loaded onto 12% SDS-polyacrylamide gels (SDS-PAGE) using low-fluorescence glass plates on an Ettan DALT II system (GE Healthcare). All electrophoresis procedures were performed in the dark. The biological triplicates were run on three gels as analytical gels. In addition, another strip was performed in parallel as a preparative gel for picking spots as described in 2-D DIGE, except that the IPG strip was loaded with 1000 mg of proteins, and the gel was stained with Coomassie brilliant blue. After SDS-PAGE, the three analytical gels were scanned with a Typhoon 9410 scanner (GE Healthcare) with appropriate excitation/emission wavelengths specific for Cy2

(488/520 nm), Cy3 (532/580 nm) and Cy5 (633/670 nm) to generate nine protein spot maps.

DeCyder 5.0 software (GE Healthcare) was used for 2-D DIGE analysis according to the manufacturer's recommendation. The DeCyder differential in-gel analysis (DIA) module was used for pairwise comparisons of each sample with the internal standard in each gel. The DeCyder biological variation analysis (BVA) module was then used to simultaneously match all nine protein spot maps, using the Cy3/Cy2 and Cy5/Cy2 DIA ratios, to calculate average abundance changes and paired Student *t* test *p* values for the variance of these ratios for each protein pair across all samples. The differential protein spots (|ratio|>2, $P<0.05$) which were altered consistently in all three protein spot maps were selected for further identification.

Immunohistochemistry (IHC)

Immunohistochemistry was performed, as previously described [2], to investigate the localization and expression of 14-3-3σ and LASP1 in 116 human CRC tissues. The sections were incubated with primary antibodies against 14-3-3σ (1:200; Sigma) and LASP1 (1:100; Millipore) overnight at 4°C. Mayer's haematoxylin was used for nuclear counterstaining. The sections were mounted with a synthetic medium. The slides were reviewed by two or three pathologists blind to the study. To evaluate 14-3-3σ and LASP1 expression levels, immunostained slides were evaluated using a method described previously [2,3]. Scores representing the percentage of positive cells were as follows: 0% (absent), 1-5% (sporadic), 6-25% (local), 26-50% (occasional), 51-75% (majority) and 76-100% (large majority). Intensity of staining of cancer cells

was scored as 0 (no staining), 1 (weak staining, light yellow), 2 (moderate staining, yellowish brown), and 3 (strong staining, brown). An intensity score of ≥ 2 with at least 50% of positive cells was considered as having high expression (or over-expression), and <50% of positive cells or < 2 in intensity score was regarded as low expression. The discrepancies (< 5%) were resolved by simultaneous re-evaluation.

Cell migration analysis

Cells from the serum-free medium (1×10^5 cells/100 μL) were added to the top chamber of each 8-mm–pore transwell chamber (Corning Star; Cambridge, Mass, USA). The bottom chamber was prepared using 10% FBS as a chemoattractant. Cells were allowed to migrate through the porous membrane for 20 h at 37°C. The cells that stuck to the lower surface of the membrane were treated with a fixation/staining solution (0.1% crystal violet, 1% formalin, and 20% ethanol) for visualization. The cells were counted under a microscope in 5 randomly selected fields (original magnification, ×200). At least 4 chambers from 3 different experiments were analyzed.

Plasmid constructs

All eukaryotic expression vectors were constructed in pEX-3 (pGCMV/MCS/Neo). Generation of 14-3-3σ was achieved by using oligonucleotides in-frame to amino-terminus of pEX-3 template by standard PCR techniques (Shanghai GenePharma Co., Shanghai, China). The coding sequence for the constructs was verified by sequencing analysis.

siRNA-mediated gene silencing

Expression of human 14-3-3σ and LASP1 was knocked down with siRNA duplexes targeting the sequence. The siRNAs were designed and chemically synthesized (Shanghai GenePharma Co., Shanghai, China) for targeting different coding regions of the genes. The negative control siRNA targeting unknown mRNA sequence was used as a control. The sequences used are shown in Table S2 (see supplementary material). Both siRNAs were synthesized from GenePharma (Shanghai, China). A BLAST search of the human genome verified that the selected sequences were specific for the target genes. Exponential growth phase cells were plated in 6-well plates at a density of 0.5×10^5 cells/ml, cultured for 24 h and transfected with 1 μg siRNA in reduced serum medium (OPTI-MEM-I) according to the manufacturer's protocol in 30-50% confluence. Fluorescein (FAM)-labeled negative control siRNA was used to visualize the transfection efficiency.

Co-immunoprecipitation (Co-IP)

The cells were transiently or stably transfected with the indicated constructs. Cells were harvested and lysed in 1 ml of lysis buffer (50 mM HEPES, 150 mM NaCl, 1 mM EDTA, 0.5% Nonidet P-40). Resulting lysates were subjected to immunoprecipitation with antibodies directed to the epitope tag. Immunoprecipitates were washed in lysis buffer, resolved by SDS-polyacrylamide gel electrophoresis, and subsequently analyzed by mass spectrum or protein immunoblotting.

Digestion of Proteins and Identification by Matrix-assisted Laser Desorption/ionization Time of Flight Mass Spectrometry

The identified protein bands in the gel were excised from the gel and were in-gel digested. Briefly, the gel bands were destained in 30 mmol/L $K_3Fe(CN)_6$ and 100 mmol/L $Na_2S_2O_3$, then dehydrated with 100% acetonitrile and dried in a stream of nitrogen gas. The dried gel pieces were incubated in a digestion solution consisting of 25 mM NH_4HCO_3 and 12.5 μg/ml trypsin (Promega, USA) for 16–18 h at 37°C. The tryptic peptide mixture was extracted and mixed with matrix a-cyano-4-hydroxycinnamic acid (CHCA) for mass spectrum analysis. Mass spectra results were obtained using an Applied Biosystems Voyager System 4800 matrix-assisted laser desorption/ionization time of flight mass spectrometry (MALDI-TOF) mass spectrometer (ABI, USA) with an accelerating voltage of MS/MS 8000 V. Mass fingerprinting was used for protein identification from tryptic fragment sizes in the NCBInr database (http://www.matrixscience.com) and SWISS-PROT database (http://web.expasy.org/docs/swiss-prot_guideline.html) with the MASCOT search engine for information such as protein name, mass score, and peptide match.

References

1 Zhao, L., Wang, H., Li, J., Liu, Y. & Ding, Y. Overexpression of Rho GDP-dissociation inhibitor alpha is associated with tumor progression and poor prognosis of colorectal cancer. *Journal of proteome research* **7**, 3994-4003, doi:10.1021/pr800271b (2008).

2 Zhao, L. *et al.* Promotion of colorectal cancer growth and metastasis by the LIM and SH3 domain protein 1. *Gut* **59**, 1226-1235, doi:10.1136/gut.2009.202739 (2010).

3 Coppola, D. *et al.* Correlation of osteopontin protein expression and pathological stage across a wide variety of tumor histologies. *Clin Cancer Res* **10**, 184-190 (2004).

Supplementary Figures

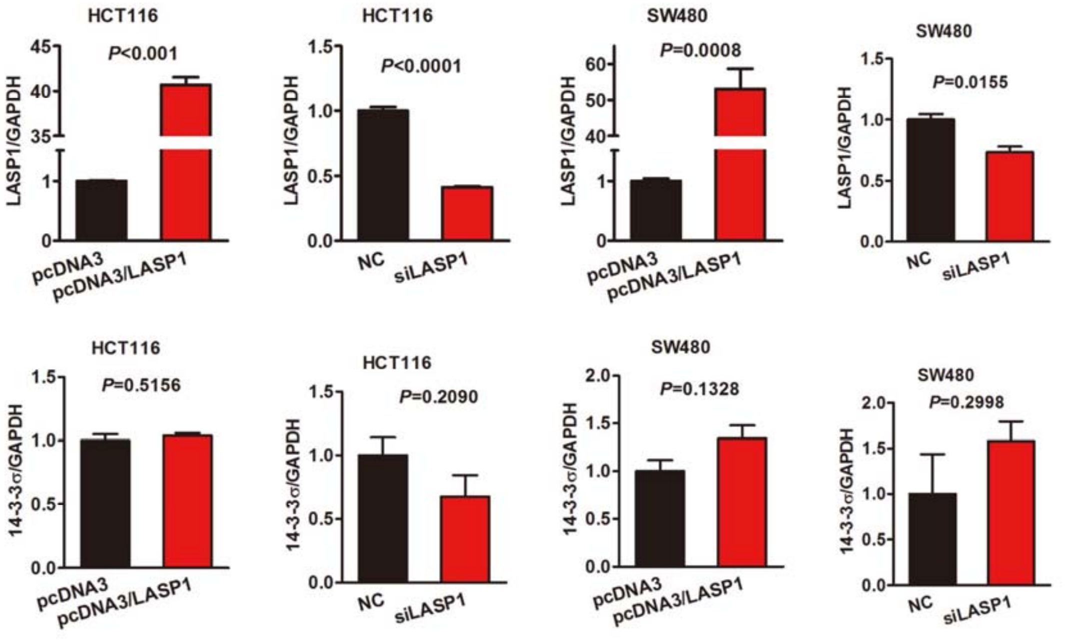

Figure S1, related to Figure 1. The effect of LASP1 expression on 14-3-3σ mRNA expression. RT-PCR assay was performed to detect the expression of 14-3-3σ and LASP1 mRNA in indicated cells.

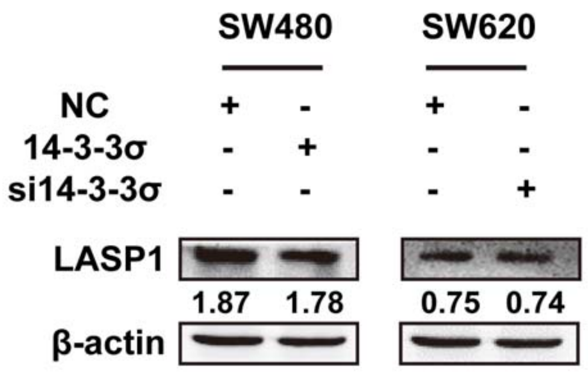

Figure S2, related to Figure 1. The effect of 14-3-3σ expression on LASP1 protein expression. Western blot was performed to detect the expression of LASP1 protein in indicated cells.

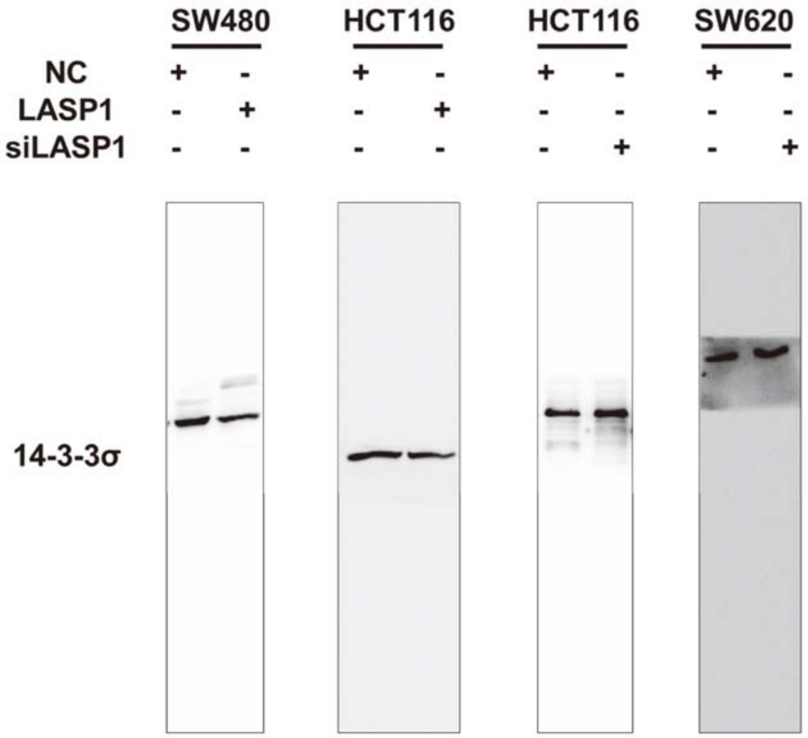

Figure S3, related to Figure 1. The full-length blots/gels including the key data presented in Fig. 1.

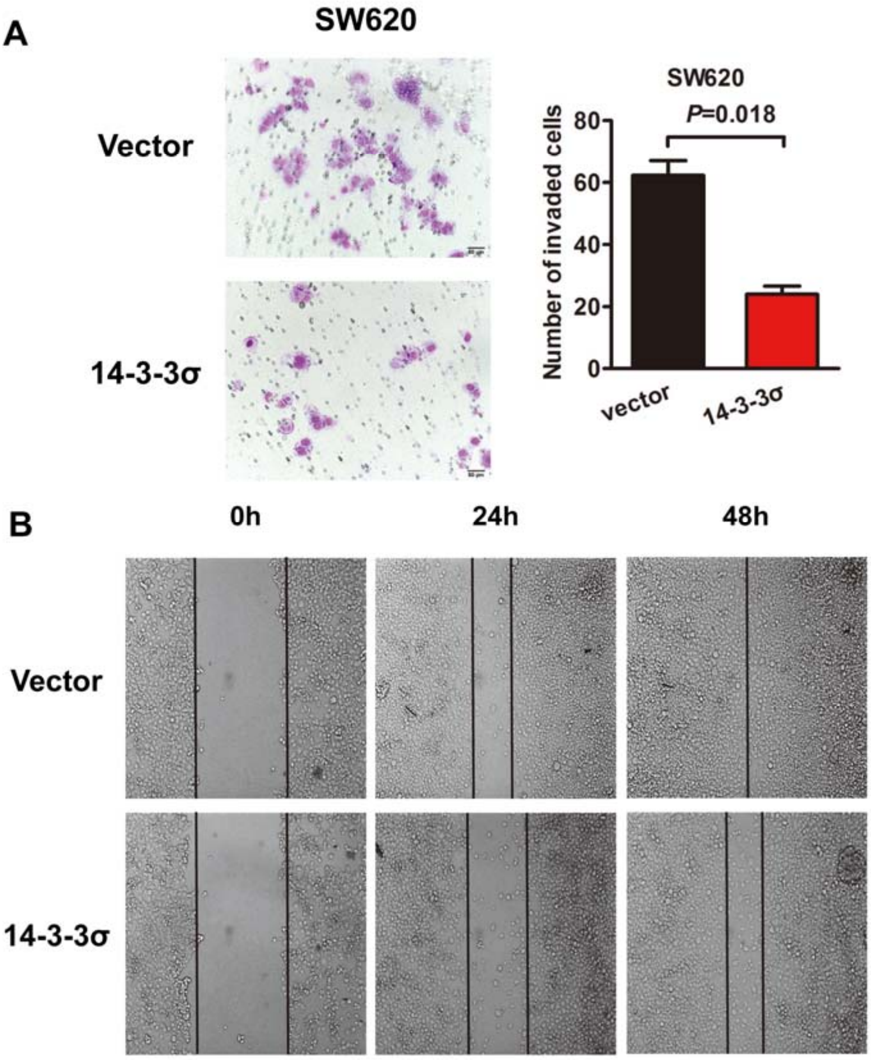

Figure S4, related to Figure 2. 14-3-3σ suppresses cell invasion and motility. (A) The representative figures and data of Matrigel-coated transwell assay for SW620 cells were transfected with 14-3-3σ vector. Each bar represented the mean ± SD. The results were reproduced in three independent experiments. (B) The representative figures of wound healing assay for SW620 cells with transfected with 14-3-3σ vector for 24h and 48h.

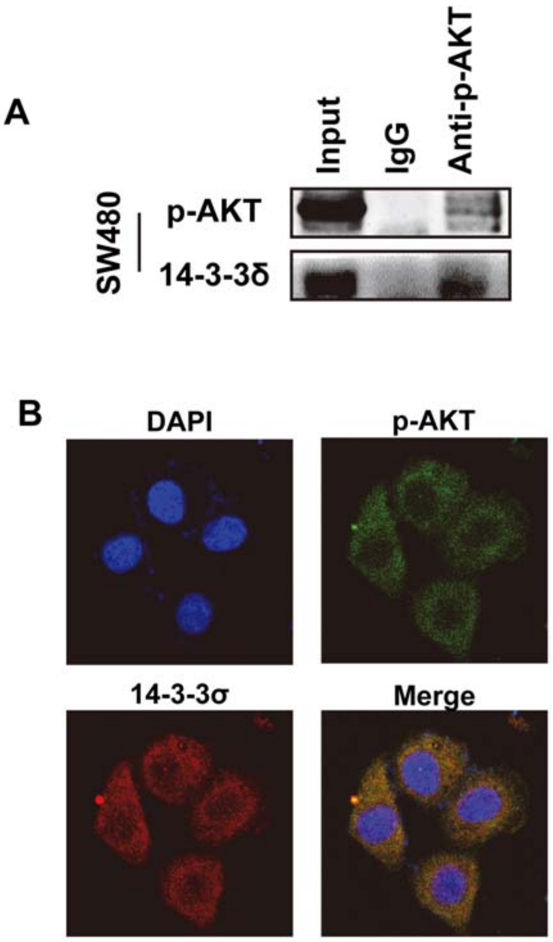

Figure S5, related to Figure 2. 14-3-3σ interacts with p-AKT. (A) Endogenous interaction between 14-3-3σ and p-AKT in SW480 cells. Cells were lysed and purified by anti-14-3-3σ affinity gel; protein pellets were analyzed by western blot with anti-p-AKT or anti-14-3-3σ. (D) The subcellular localization of 14-3-3σ and p-AKT in SW480 cells was assessed by immunofluorescence staining.

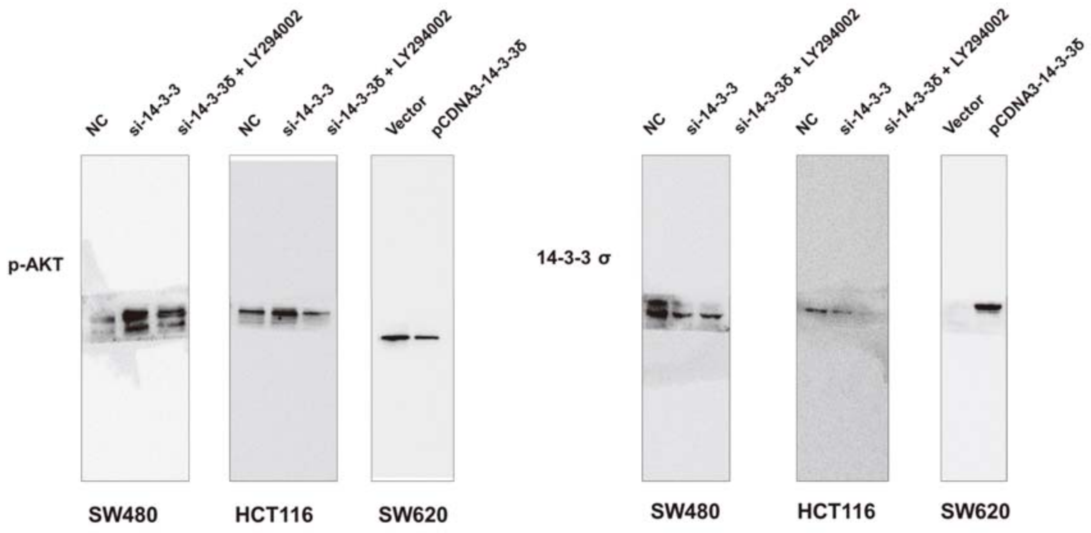

Figure S6, related to Figure 2. The full-length blots/gels including the key data presented in Fig. 2.

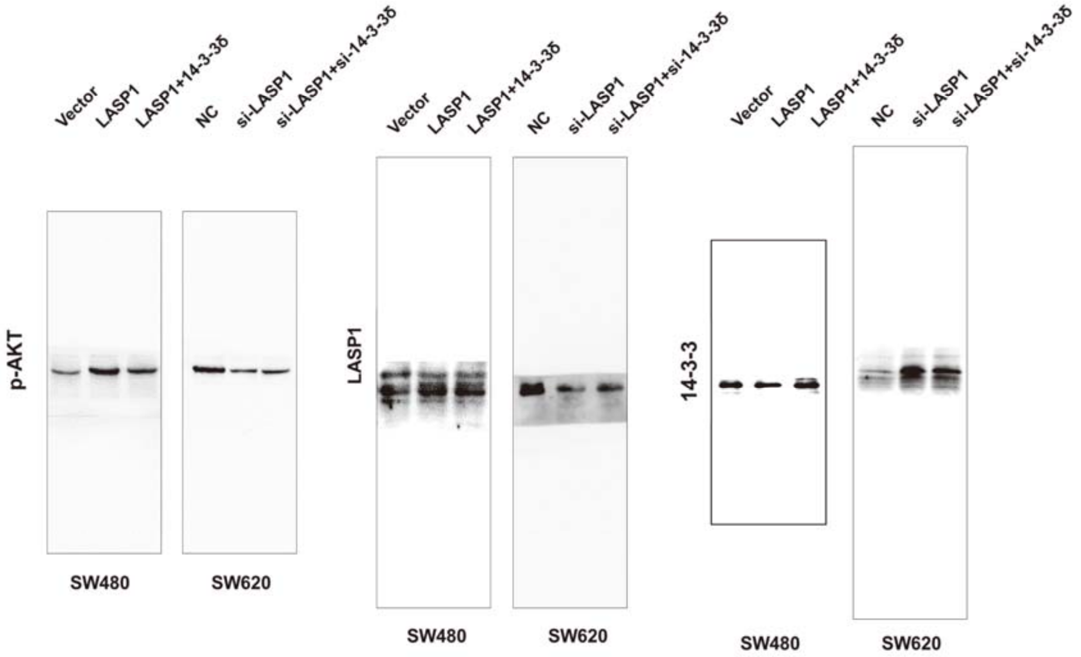

Figure S7, related to Figure 3. The full-length blots/gels including the key data presented in Fig. 3.

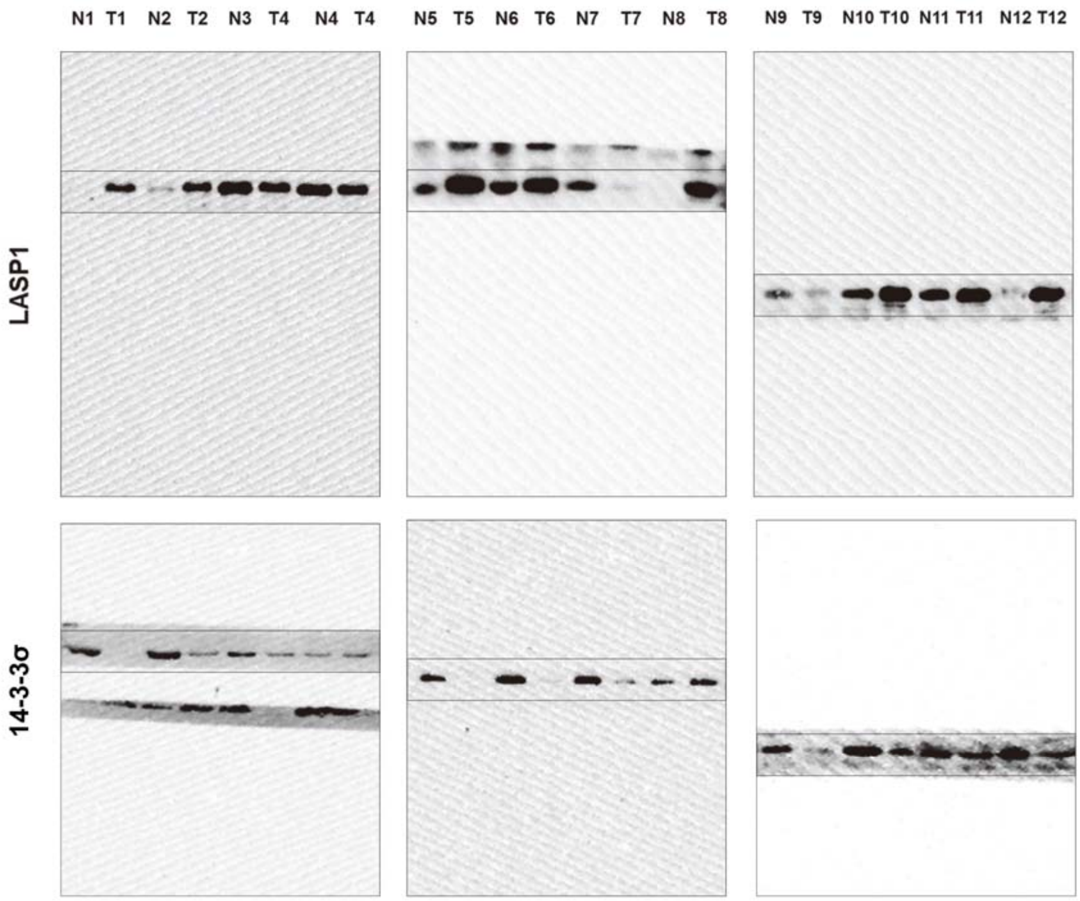

Figure S8, related to Figure 5. The full-length blots/gels including the key data presented in Fig. 5.

Supplementary Table S1. RT-PCR primer sequences for human genes

Gene	Forward primer	Reverse primer	Product length
**14-3-3δ**	CTGAGCTCCAGGGCGTGTGC	CCTGGTAGGCTGACCGGGCT	189bp
**LASP1**	GCAACAGAGTGAGCTCCAGAG	TGAAACCTTTGCCCTTGTTC	70bp
**GAPDH**	GGAGCGAGATCCCTCCAAAAT	GGCTGTTGTCATACTTCTCATGG	197bp

Supplementary Table S2. siRNA sequences used for transfection

Gene name	Sense 5' to 3'	Antisense 5' to 3'
**LASP1**	UUCUCCGAACGUGUCACGUTT	ACGUGACACGUUCGGAGAATT
**14-3-3δ**	UGAAGAUGAAGGGUGACUATT	UAGUCACCCUUCAUCUUCATT
**Negative control (NC)**	UUCUCCGAACGUGUCACGUTT	ACGUGACACGUUCGGAGAATT

Supplementary Table S3. Correlation between the clinicopathological features and 14-3-3σ expression

Characteristics	14-3-3δ expression					
	Negative	Positive	*P* value	Low	High	*P* value
Normal	4	48	0.433	15	37	0.011*
Cancer	15	101		58	58	
**Gender**						
Male	8	63	0.502	36	35	0.849
Female	7	38		22	23	
**Age(years)**						
<50	1	16	0.894	8	9	0.793
≥50	14	85		50	49	
**Tumor site**						
Colon	5	50	0.242	28	27	0.852
Rectum	10	51		30	31	
**Tumor size (cm in diameter)**						
<5	6	58	0.205	30	34	0.455

≥5	9	43		28	24	
**Differentiation**						
Good/Moderate	14	87	0.689	47	54	0.094
Poor	1	14		11	4	
**T classification**						
T1+T2	0	13	0.213	4	9	0.238
T3+T4	15	88		54	49	
**N classification**						
N0	7	58	0.433	24	41	0.001*
N1+N2	8	43		34	17	
**M classification**						
M0	13	92	0.633	49	56	0.053
M1	2	9		9	2	
**LASP1 expression**						
Low	6	50	0.492	13	43	0.000*
High	9	51		45	15	

* Statistically significant ($P < 0.05$).

Supplementary Table S4. Univariate and multivariate analyses of individual parameters for correlations with overall survival rate: Cox proportional hazards model

**Variables**	**Univariate**			**Multivariate**		
	**OR**	**CI(95%)**	***P* value**	**OR**	**CI(95%)**	***P* value**
**Gender**	0.408	0.194-0.858	0.018*	0.446	0.211-0.944	0.035*
**Age**	1.791	0.637-5.034	0.269			
**Tumor site**	1.101	0.805-1.507	0.546			
**Tumor size**	1.544	0.830-2.873	0.170			
**Differentiation**	0.299	0.145-0.615	0.001*	0.472	0.221-1.011	0.053
**T classification**	3.035	0.732-12.592	0.126			
**N classification**	4.535	2.261-9.096	0.000*	2.457	1.151-5.246	0.020*
**M classification**	7.729	3.699-16.146	0.000*	3.605	1.643-7.910	0.001*
**Low 14-3-3δ expression**	0.254	0.124-0.522	0.000*	0.419	0.192-0.912	0.028*

Abbreviations: OR, Odds ratio; CI, Confidence interval.

* Statistically significant ($P < 0.05$).
